# Comparison between long and short-term venous patencies after pancreatoduodenectomy or total pancreatectomy with portal/superior mesenteric vein resection stratified by reconstruction type

**DOI:** 10.1371/journal.pone.0240737

**Published:** 2020-11-05

**Authors:** Kai Siang Chan, Nandhini Srinivasan, Ye Xin Koh, Ek Khoon Tan, Jin Yao Teo, Ser Yee Lee, Peng Chung Cheow, Prema Raj Jeyaraj, Pierce Kah Hoe Chow, London Lucien Peng Jin Ooi, Chung Yip Chan, Alexander Yaw Fui Chung, Brian Kim Poh Goh

**Affiliations:** 1 Department of Hepatopancreatobiliary and Transplant Surgery, Singapore General Hospital, Singapore, Singapore; 2 Singapore Health Services Pte Ltd, Singapore, Singapore; 3 Duke-National University of Singapore Medical School Singapore, Singapore, Singapore; Ohio State University Wexner Medical Center Department of Surgery, UNITED STATES

## Abstract

**Background:**

Venous reconstruction has been recently demonstrated to be safe for tumours with invasion into portal vein and/or superior mesenteric vein. This study aims to compare the patency between various venous reconstructions.

**Methods:**

This is retrospective study of 76 patients who underwent pancreaticoduodenectomy or total pancreatectomy with venous reconstruction from 2006 to 2018. Patient demographics, tumour histopathology, morbidity, mortality and patency were studied. Kaplan-Meier estimates were performed for primary venous patency.

**Results:**

Sixty-two patients underwent pancreaticoduodenectomy and 14 underwent total pancreatectomy. Forty-seven, 19 and 10 patients underwent primary repair, end-to-end anastomosis and interposition graft respectively. Major morbidity (Clavien-Dindo >grade 2) and 30-day mortality were 14/76(18.4%) and 1/76(1.3%) respectively. There were 12(15.8%) venous occlusion including 4(5.3%) acute occlusions. Overall 6-month, 1-year and 2-year primary patency was 89.1%, 92.5% and 92.3% respectively. 1-year primary patency of primary repair was superior to end-to-end anastomosis and interposition graft (primary repair 100%, end-to-end anastomosis 81.8%, interposition graft 66.7%, p = 0.045). Pairwise comparison also demonstrated superior 1-year patency of primary repair (adjusted p = 0.037). There was no significant difference between the cumulative venous patency for each venous reconstruction method: primary repair 84±6%, end-to-end anastomosis 75±11% and interposition graft 76±15% (p = 0.561).

**Conclusion:**

1-year primary venous patency of primary repair is superior to end-to-end anastomosis and interposition graft.

## 1. Introduction

Pancreatic ductal adenocarcinoma (PDAC) is the most common pancreatic neoplasm with 5-year survival as low as 6% [1, 2]. The low 5-year survival rate is often due to delayed diagnosis and only 20% of patients are eligible for surgical resection at presentation[2]. Resection of PDAC of the pancreatic head is often complicated by tumour adherence to the portal vein (PV) or superior mesenteric vein (SMV) due to the its close anatomical relationship [3]. In the past, this was considered a contraindication for curative pancreatic surgery, but more recent studies have shown that combined resection of the pancreas, PV and SMV had comparable morbidity, mortality and survival as pancreatic surgery without venous resection [4–8]. An expert consensus in 2009 by Evans et al. recommended the use of pancreaticoduodenectomy with venous resection and/or venous reconstruction as a standard of practice for PDAC with local invasion into PV, SMV, and/or PV-SMV confluence in experienced institutions [9].

Several techniques have been reported for venous reconstruction including: (1) primary lateral venorrhaphy (2) primary end-to-end anastomosis (3) primary repair with venous patch (4) interposition graft with synthetic or autologous graft [10]. Presently, there is a paucity of studies comparing the patency or thrombosis rates between various venous reconstruction methods. Overall rates of occlusion of PV has been reported to range between 0–17% and is dependent on: (1) extent of resection (2) method of reconstruction (3) timing and mode of graft surveillance [11–17]. Some investigators have reported that polytetrafluoroethylene (PTFE) interposition grafts may have a higher incidence of thrombosis but results remain inconclusive due to small sample size [15]. The primary objective of the present study was to investigate and compare the patency rates between the various methods of venous reconstruction after pancreaticoduodenectomy and total pancreatectomy. The secondary aim was to compare the difference in perioperative outcomes, morbidity and mortality across the various types of venous reconstruction.

## 2. Materials and methods

This is a single institution retrospective study of all consecutive patients who underwent pancreaticoduodenectomy or total pancreatectomy with venous resection and reconstruction from 2006 to 2018 at Singapore General Hospital. This study was approved by the SingHealth Centralised Institutional Review Board (Ref number: 2020/2066) on 19 February 2020. All patients were identified from our prospective pancreatic resection database. Patients with concomitant arterial reconstruction were excluded from the study. Patient demographics and study variables were extracted from computerized clinical databases. Patients were stratified into 3 groups according to the reconstruction type: primary repair, primary end-to-end anastomosis and interposition graft. All data were fully anonymized prior to access by the study team. The patients’ medical records were accessed from January 2006 to December 2018.

### 2.1 Study variables and outcomes

The study variables were age, gender, American Society of Anaesthesiology (ASA) score, type of venous resection, histopathological findings and tumour size. Study outcomes were need for blood transfusion, intensive care unit (ICU) admission and reoperation, length of hospitalisation stay, tumour recurrence, follow-up duration, patency rates (≤30 days and >30 days, and primary patency vs secondary patency), morbidity (all morbidity and ≥grade 3 on Clavien-Dindo grading system) and mortality (30-day, 90-day and in-hospital mortality) [18]. Postoperative pancreatic fistula (POPF) was defined and graded according to the latest International Study Group for Pancreatic Fistula classification system [19].

### 2.2 Definitions

Primary repair was defined as either transverse or lateral venorrhaphy. Patch repair was not included under primary repair. Patency was defined as presence of hepatopetal flow on ultrasound doppler or venous opacification with contrast on computed tomography (CT) scan. Partial occlusions such as focal narrowing of veins or presence of stable partial thrombus were considered to be patent. Patency was expressed as a percentage over the number of patients who were still alive at the respective time intervals. Occlusion was defined as complete occlusion such as absence of hepatopetal flow, presence of complete thrombus or cavernous transformation. Primary patency was defined as patency after the initial venous reconstruction; secondary patency was defined as patency following an invasive intervention (thrombectomy or revision surgery with reconstruction). Patients who did not receive any invasive intervention after primary occlusion were also considered to be non-patent under secondary patency.

All morbidity was defined as the presence of any morbidity, and major morbidity was defined as the presence of any complications of ≥grade 3 on the Clavien-Dindo grading system. Thirty-day and 90- was defined as any death within 30 days and 90 days after surgery respectively. In-hospital mortality was defined as any death during the index hospital stay regardless of time from surgery. Disease-specific survival was defined as patients who have not died from the underlying disease and excluded all patients with 90-day mortality.

### 2.3 Treatment protocol

All patients who underwent pancreaticoduodenectomy and total pancreatectomy had histopathological analyses of the specimens. Two intra-abdominal drains are placed and drain fluid amylase were analysed on postoperative day 1, 3 and 5. Prophylactic antibiotics were administered to all patients undergoing surgery and continued post-operatively based on the clinical status of the patients. Somatostatin infusion was given to selected group of patients with higher risk of pancreatic fistula formation over 5 days [20–22]. Duration of ICU and/or high dependency unit stay was dependent on clinical status of the patient. Patients at high risk of developing thromboses, or had clinical symptoms and/or signs of early PV and/or SMV thrombosis or occlusion underwent doppler ultrasound to look for patency. All patients had routine follow-up at the specialist outpatient clinic at 3 months, 6 months, 1 year and thereafter annually, with routine doppler ultrasound and/or CT with contrast to look for any PV and/or SMV occlusion. Reduced patency of PV and/or SMV were managed based on the extent of occlusion: (1) monitoring for progression of occlusion (2) medical management with low molecular weight heparin and/or anti-platelets. Decision to start anti-coagulation was made based on a shared decision between surgeon and patients.

### 2.4 Statistical analysis

All the data extracted were extracted from the department REDCap database and tabulated into SPSS version 25 (SPSS, SPSS inc, Chicago IL, USA) for statistical analysis. Shapiro-Wilk test of normality was performed. Categorical values were described as percentage and analysed by chi-square test or Fisher’s exact test as appropriate. Continuous variables were expressed as median and analysed by Kruskal-Wallis test. Cumulative primary venous patency and survival were analysed using Kaplan-Meier estimates with life table analysis. Log-rank test was performed to determine statistical significance between the cumulative estimates. Statistical significance was defined as p<0.05. Pairwise comparison was performed using Bonferroni correction for variables with statistically significant differences.

## 3. Results

A total of 76 patients were included in the study: 62 patients underwent pancreaticoduodenectomy and 14 patients underwent total pancreatectomy with venous reconstruction. Methods of venous reconstruction included primary repair (n = 47/76, 61.8%), end-to-end anastomosis (n = 19/76, 25.0%) and interposition graft (n = 10/76, 13.2%). One of the patients underwent initial primary end-to-end anastomosis, followed by interposition graft reconstruction as a revision surgery due to acute thrombosis. The patient was reported to have undergone primary end-to-end anastomosis, which was the initial intervention performed with intention to treat.

### 3.1 Clinicopathological profile

Table 1 demonstrates the clinicopathological profile of all the patients who underwent either pancreaticoduodenectomy or total pancreatectomy with venous reconstruction. Table 2 shows the perioperative details of all patients. Forty-one out of 76(53.9%) patients experienced post-operative complications: cardiovascular (n = 11/76, 14.5%), respiratory (n = 13/76, 17.1%), delayed gastric emptying (n = 20/76, 26.3%), POPF International Study Group on Pancreatic Fistula Definition grade B or C (n = 5/76, 6.6%) and post-pancreatectomy haemorrhage (n = 8/76, 10.5%).

**Table 1:** Clinicopathological features profile of all patients who underwent pancreaticoduodenectomy or total pancreatectomy with venous reconstruction.

	All (n = 76)	Primary repair (n = 47)	Primary end-to-end anastomosis (n = 19)	Interposition graft (n = 10)	*p* value
Median age (IQR)	66 (58.5–70.8)	65 (54–70)	67 (63–73)	63.5 (52.5–70.3)	0.449
Gender, male, n (%)	36 (47.4)	24 (51.1)	9 (47.4)	3 (30)	0.480
ASA score (IQR)	2 (2–3)	2 (2–3)	2 (2–2)	2 (2–2.3)	0.889
Tumour size, cm (IQR)	3.5 (2.7–4.4)	3.5 (2.6–4.0)	3.5 (2.3–4.0)	4.3 (3.6–5.8)	0.104
Malignant tumour, n (%)	73 (96.1)	46 (97.9)	18 (94.7)	9 (90)	0.481
Histopathology					0.383
PDAC	62 (81.6)	40 (85.1)	16 (84.2)	6 (60)	
Neuroendocrine tumour	3 (3.9)	2 (4.3)	1 (5.3)	0 (0)	
Cholangiocarcinoma	1 (1.3)	1 (2.1)	0 (0)	0 (0)	
IPMN	2 (2.6)	0 (0)	1 (5.3)	1 (10)	
Others	5 (6.6)	3 (3.9)	0 (0)	2 (20)	
Benign	3 (3.9)	1 (2.1)	1 (5.3)	1 (10)	

All continuous variables are expressed in median unless otherwise specified.

ASA: American Society of Anaesthesiology, IPMN: Intraductal papillary mucinous neoplasm, IQR: Interquartile range, PDAC: Pancreatic ductal adenocarcinoma.

**Table 2:** Perioperative details of all patients who underwent venous reconstruction.

	All (n = 76)	Primary repair (n = 47)	Primary end-to-end anastomosis (n = 19)	Interposition graft (n = 10)	*p* value
Type of operation					0.083
Pancreaticoduodenectomy, n (%)	62 (81.6)	42 (89.4)	13 (68.4)	7 (70)	
Total pancreatectomy, n (%)	14 (18.4)	5 (10.6)	6 (31.6)	3 (30)	
Operating time, min (IQR)	465 (391–549)	435 (360–515)	480 (400–510)	580 (534–611)	**0.002**
Blood transfusion, n (%)	21 (27.6)	13 (27.7)	2 (10.5)	6 (60)	**0.018**
Vein resected					0.780
PV	26 (34.2)	16 (34)	7 (36.8)	3 (30)	
SMV	37 (48.7)	23 (48.9)	10 (52.6)	4 (40)	
PV/SMV confluence	13 (17.1)	8 (17)	2 (10.5)	3 (30)	
All morbidity, n (%)	41 (53.9)	26 (55.3)	11 (57.9)	4 (40)	0.626
Major morbidity (≥ Grade 3), n (%)	14 (18.4)	9 (19.1)	2 (10.5)	3 (30)	0.428
Reoperation, n (%)	10 (13.2)	6 (12.8)	2 (10.5)	2 (20)	0.767
ICU stay, n (%)	27 (35.5)	16 (34)	5 (26.3)	6 (60)	0.186
Length of stay, days (IQR)	14 (10–27)	12 (10–26)	18 (11–42)	14 (9–42)	0.214
Mortality					
30-day mortality, n (%)	1 (1.3)	1 (2.1)	0 (0)	0 (0)	0.732
90-day mortality, n (%)	4 (5.3)	2 (4.3)	0 (0)	2 (20)	0.064
In-hospital mortality, n (%)	3 (3.9)	2 (4.3)	0 (0)	1 (10)	0.415
PV and/or SMV occlusion, n (%)					
30 days, n (%)	4 (5.3)	1 (2.1)	2 (10.5)	1 (10)	0.296
90 days, n (%)	5 (6.6)	2 (4.3)	2 (10.5)	1 (10)	0.581
180 days, n (%)	7 (9.2)	3 (6.4)	2 (10.5)	2 (20)	0.390
Adjuvant chemotherapy, n (%)	49 (64.5)	33 (70.2)	11 (57.9)	5 (50)	0.377
Adjuvant radiotherapy, n (%)	9 (11.8)	5 (10.6)	3 (15.8)	1 (10)	0.826
Tumour recurrence, yes (%)	46 (60.5)	28 (59.6)	14 (73.7)	4 (40)	0.206
Length of follow-up, months (IQR)	9.1 (5.7–17.7)	9.0 (6.0–17.9)	9.9 (6.4–20.1)	5.3 (2.1–12.7)	0.152
Median length of survival, months (IQR)	9.2 (6.7–19.6)	10.1 (7.0–23.8)	9.9 (6.9–18.8)	6.9 (2.8–11.0)	0.181

All continuous variables are expressed in median unless otherwise specified.

ICU: Intensive care unit, IQR: Interquartile range, PV: Portal vein, SMV: Superior mesenteric vein.

### 3.2 Venous patency

Twelve out of 76(15.8%) patients had venous occlusion. The overall 6-month, 1-year and 2-year primary venous patency was 89.1%, 92.5% and 92.3% respectively (Table 3), with a median length to follow-up of 9.1 months (IQR 5.7–15.7). 4 out of 76 patients (5.3%) patients had acute occlusion (≤30 days): all of them underwent thrombectomy with a fogarty catheter.

**Table 3:** Venous patency of patients who underwent venous reconstruction in pancreaticoduodenectomy or total pancreatectomy.

	All	Primary repair	Primary end-to-end anastomosis	Interposition graft	*p* value
Primary patency, yes					
30-day	71/75 (94.7)	45/46 (97.8)	17/19 (89.5)	9/10 (90)	0.196
3-month	67/72 (93.1)	43/45 (95.6)	17/19 (89.5)	7/8 (87.5)	0.470
6-month	57/64 (89.1)	37/40 (92.5)	15/17 (88.2)	5/7 (71.4)	0.257
1-year	37/40 (92.5)	26/26 (100)	9/11 (81.8)	2/3 (66.7)	**0.045**
2-year	24/26 (92.3)	18/18 (100)	4/5 (80)	2/3 (66.7)	0.086
3-year	18/20 (90)	14/14 (100)	3/4 (75)	1/2 (50)	0.079
Secondary patency, yes					
30-day	73/75 (97.3)	45/46 (97.8)	18/19 (94.7)	10/10 (100)	0.627
3-month	67/72 (93.1)	43/45 (95.6)	17/19 (89.5)	7/8 (87.5)	0.470
6-month	57/64 (89.1)	37/40 (92.5)	15/17 (88.2)	5/7 (71.4)	0.257
1-year	37/40 (92.5)	26/26 (100)	9/11 (81.8)	2/3 (66.7)	**0.045**
2-year	24/26 (92.3)	18/18 (100)	4/5 (80)	2/3 (66.7)	0.086
3-year	18/20 (90)	14/14 (100)	3/4 (75)	1/2 (50)	0.079

Primary venous patency was significantly different between the various reconstruction methods at 1-year (primary repair 100%, end-to-end anastomosis 81.8%, interposition graft 66.7%, p = 0.045). Pairwise comparison demonstrated superior 1-year patency of primary repair over primary end-to-end anastomosis and interposition graft (adjusted p = 0.037). Secondary venous patency showed similar superiority in 1-year patency of primary repair over other methods.

### 3.3 Patients with acute occlusion (≤30 days)

Four patients had acute occlusion within 30 days. Patient A underwent a 2-cm segmental resection of the SMV with primary end-to-end anastomosis. A 4-cm thrombus in the SMV was noted 2 days later and underwent thrombectomy with fogarty balloon. Patient was also started on intravenous heparin. Ultrasound doppler showed good venous flow. A 5-cm thrombus was subsequently noted extending from the bifurcation of SMV and decision was made for SMV resection with reconstruction using a 8mm PTFE graft. Patency was confirmed using ultrasound doppler. The patient subsequently underwent an explorative laparotomy for gastrojejunal dehiscence likely secondary to the SMV thrombus. Patient B initially underwent a SMV reconstruction with common femoral vein graft; SMV thrombosis was noted and thrombectomy using fogarty balloon was performed. Patient C underwent segmental SMV resection with primary repair and subsequent SMV thrombectomy with fogarty balloon. The patient underwent laparotomy washout due to bilious content with high amylase in abdominal drain. Patient D underwent SMV resection with primary end-to-end anastomosis. PV thrombus was noted subsequently and the patient underwent thrombectomy with fogarty balloon with heparin infusion. Explorative laparotomy was subsequently performed in view of possible gastrojejunal leak. None of the abovementioned patients died from the acute occlusion and subsequent complications.

### 3.4 Patients who underwent interposition graft

Ten patients underwent venous reconstruction with interposition graft (excluding the patient underwent revision surgery): 6(60%) had PTFE graft (1 of them had both a PTFE graft and vein allograft) and 4(40%) had autologous graft (common femoral vein or great saphenous vein graft). Primary patency for PTFE graft was 100% at 30-day, 6-month, 1-year and 2-year, while patency for vein allograft was 75% at 30-day, 50% at 6-month and 0% at 1-year and 2-year (Table 4). There were no statistically significant differences. None of the patients experienced graft infection.

**Table 4:** Primary venous patency of patients who underwent interposition graft reconstruction.

	All	PTFE graft	Vein allograft	*p* value
30-day, n (%)	9/10 (90)	6/6 (100)	3/4 (75)	0.400
3-month, n (%)	6/7 (85.7)	4/4 (100)	3/4 (75)	1.000
6-month, n (%)	5/7 (71.4)	3/3 (100)	2/4 (50)	0.429
1-year, n (%)	2/3 (66.7)	2/2 (100)	0/1 (0)	0.333
2-year, n (%)	2/3 (66.7)	2/2 (100)	0/1 (0)	0.333
3-year, n (%)	1/2 (50)	1/1 (100)	0/1 (0)	1.000

PTFE: Polytetrafluoroethylene.

**Fig 1:**
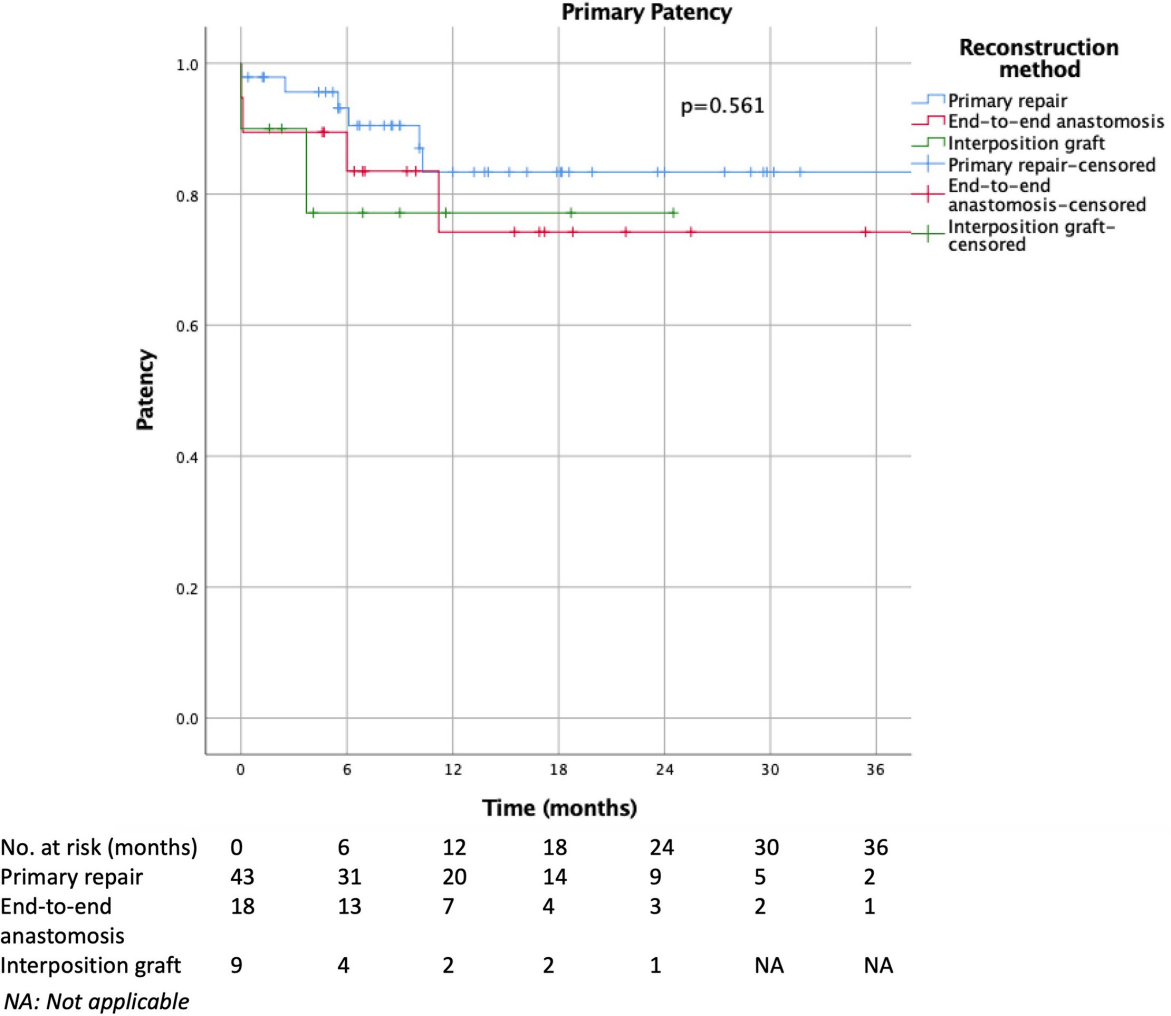
Comparison of primary patency rates among primary repair, primary end-to-end anastomosis and interposition graft.

Using the Kaplan-Meier and life table estimates, the cumulative overall 2-year venous patency was 80±5%. Cumulative venous patency was the same at 6 months, 1 year and 2 year for each VR method (Fig 1): 84±6% for primary repair, 75±11% for primary end-to-end anastomosis, 76±15% for interposition graft (p = 0.561). Pairwise comparison was also similar: primary repair vs interposition graft p = 0.343, primary end-to-end anastomosis vs interposition graft p = 0.868, primary repair vs end-to-end anastomosis p = 0.404. Comparison of primary repair with combined primary end-to-end anastomosis and interposition graft also did not reveal any statistical significance (p = 0.299).

### 3.5 Mortality

**Fig 2:**
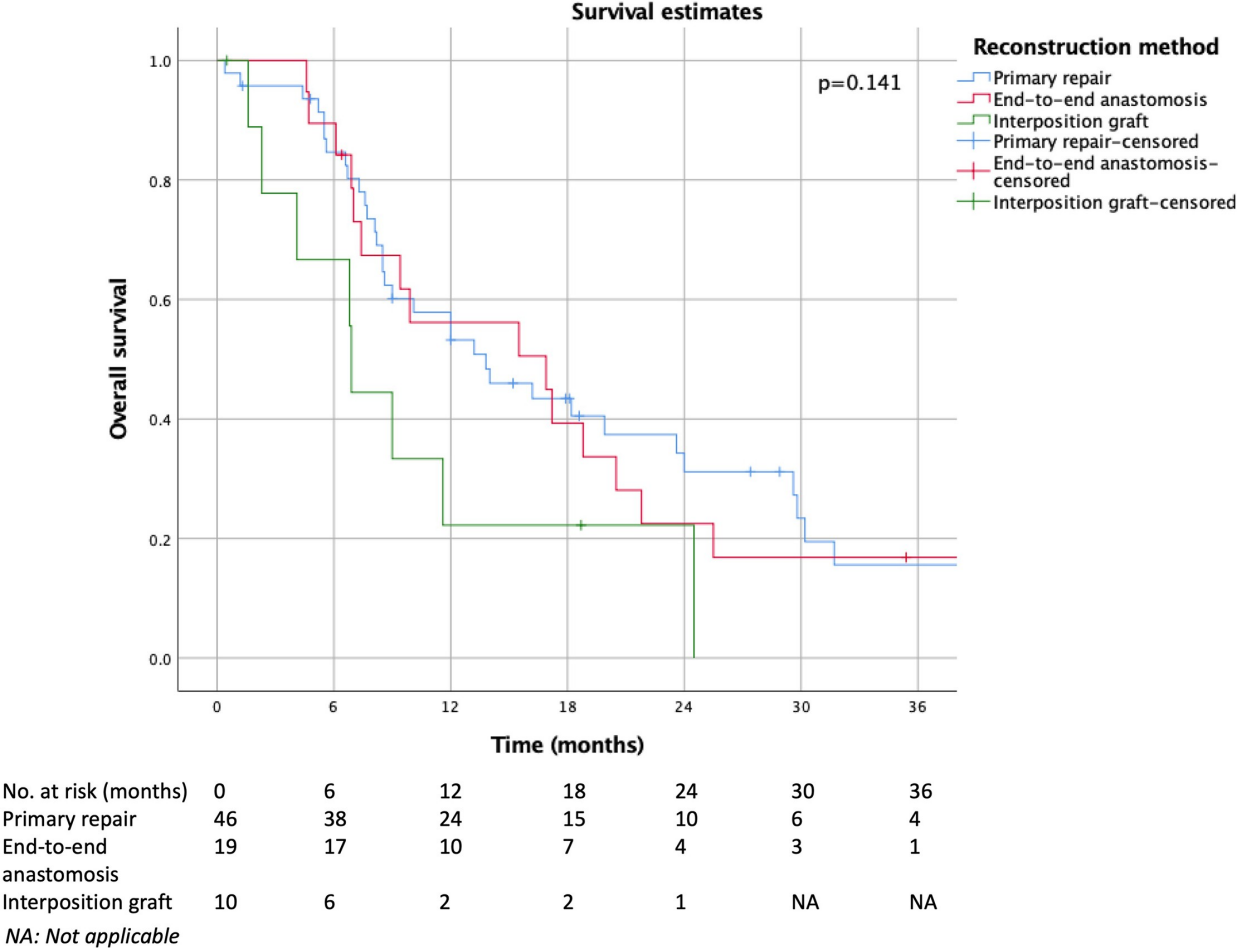
Comparison of overall survival among primary repair, primary end-to-end anastomosis and interposition graft.

Table 2 demonstrates the 30-day, 90-day and in-hospital mortality across various venous reconstruction methods. Kaplan-Meier and life table estimates showed no significant differences in cumulative survival estimates (p = 0.141) (Fig 2). Pairwise comparison did not demonstrate any difference in survival between interposition graft vs primary repair (p = 0.053) and interposition graft vs end-to-end anastomosis (p = 0.117). Cumulative 1-year survival estimates for primary repair, end-to-end anastomosis and interposition graft was 43±8%, 40±12% and 23±14% respectively. Median estimated length of survival was 15.1 months for primary repair, 14.4 months for end-to-end anastomosis and 8.4 months for interposition graft.

**Fig 3:**
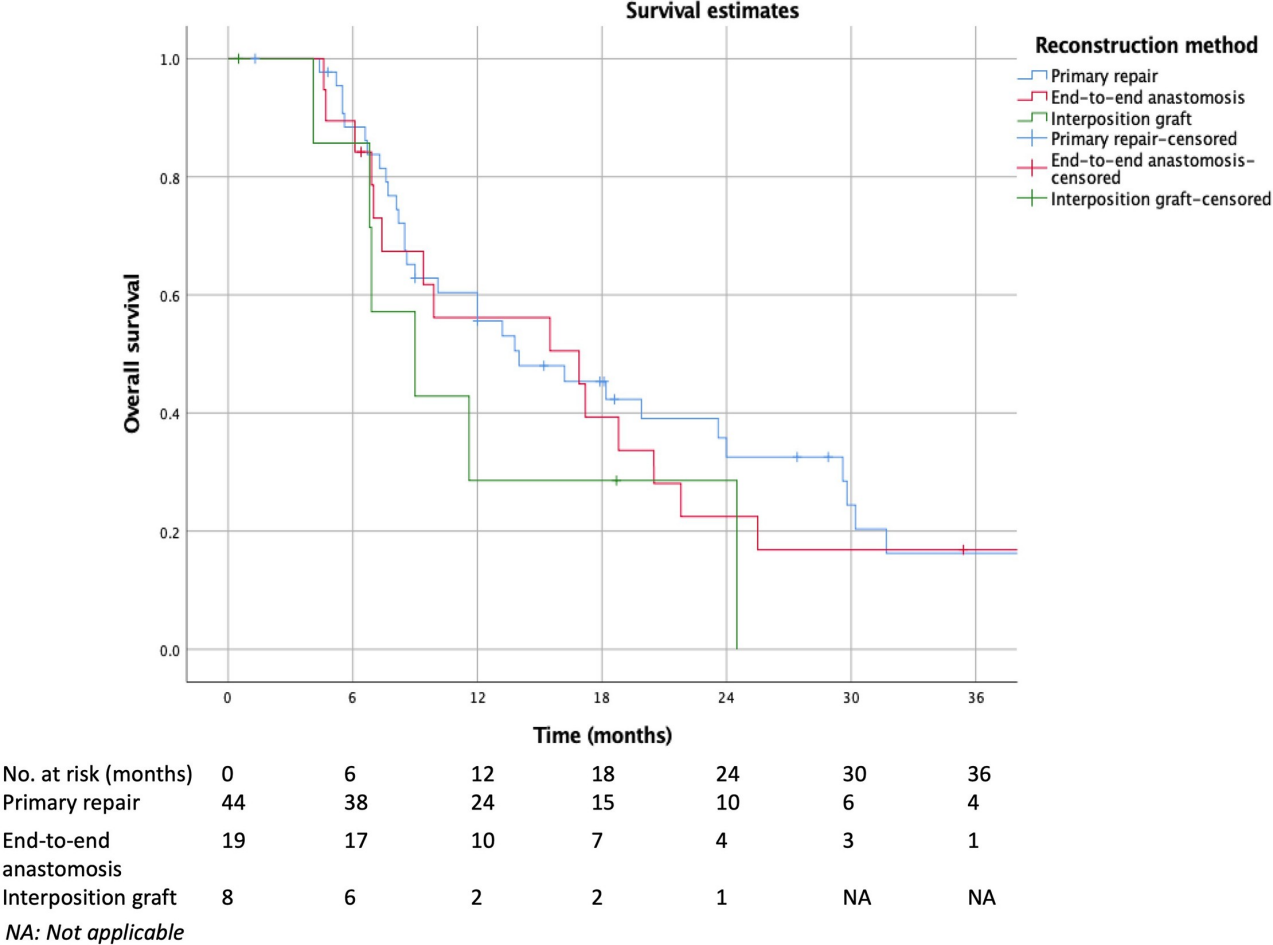
Comparison of disease-specific survival among primary repair, primary end-to-end anastomosis and interposition graft.

Kaplan-Meier and life table estimates for disease-specific survival also did not show any significant differences in cumulative estimates (p = 0.446)(Fig 3). Cumulative 1-year survival estimates for primary repair, end-to-end anastomosis and interposition graft was 45±8%, 40±12% and 29±17% respectively. Median estimated length of survival was 16.0 months for primary repair, 14.4 months for end-to-end anastomosis and 9.8 months for interposition graft.

## 4. Discussion

The present study demonstrates superior patency of primary repair over end-to-end anastomosis and interposition graft in pancreaticoduodenectomy or total pancreatectomy. To date, there is a paucity of literature on the patency rates of various methods of venous reconstruction in pancreatectomy. A meta-analysis by Gao et al. which analysed 1906 patients in 29 retrospective studies demonstrated superior long-term patency rates of primary repair over synthetic interposition graft (Odds ratio (OR): 2.32, 95% confidence interval (CI): 1.25–4.31, p = 0.008), but showed similar patency rates between primary repair and autologous grafting (OR: 0.93, 95% CI: 0.53–1.63, p = 0.79) [23]. This study aims to contribute to the existing literature by comparing the patency of various methods of venous reconstruction in pancreaticoduodenectomy and/or total pancreatectomy.

Cumulative primary patency estimates using Kaplan-Meier curve estimates did not reveal any statistically significant differences across the types of venous reconstruction (1-year cumulative patency for primary repair 84%, end-to-end anastomosis 75%, interposition graft 76%, p = 0.561). This finding was consistent with the study by Liao et al. [24]; Liao et al demonstrated similar estimated cumulative patency at 1-year between PTFE graft and primary anastomosis (83.5% and 86.4% respectively). The lack of statistical significance in both the study by Liao et al and our present study may be due to the small denominator contributing to a type 2 error. This may also be due to the short length of follow-up (median 9.1 months) and length of survival (median 9.2 months) in the present study cohort.

Interposition graft is usually only considered when primary repair or end-to-end anastomosis is contraindicated, such as a long resection length due to tumor involvement which may result in too much tension for primary anastomosis [25]. Our study demonstrated high primary venous patencies with a 30-day patency of 94.7% and 2-year patency of 92.3%. This is consistent with existing studies which demonstrate high patency rates after pancreatectomy with venous reconstruction [26]. A study by Dua et al. in 2015 demonstrated superior 1000-day patency of primary end-to-end anastomosis and longitudinal venorrhaphy over other venous reconstruction methods (end-to-end anastomosis and transverse venorrhaphy 100%, longitudinal venorrhaphy 71.3%, IG 63.2%) [27]. This is inconsistent with our study, which demonstrates comparable 3-year patency (primary repair 100%, end-to-end anastomosis 75%, interposition graft 50%, p = 0.079). This may be due to the different sample population between our studies: our study included both longitudinal and transverse venorrhaphy under primary repair. Nevertheless, our studies demonstrate lower patency of interposition graft over other methods. A recent study in 2019 by Terasaki et al. which compared end-to-end anastomosis with interposition graft demonstrated no significant differences in portal vein stenosis at 6 months (end-to-end anastomosis 6%, interposition graft 4%, p = 0.561) and 1 year (end-to-end anastomosis 14%, interposition graft 16%, p = 0.529) after surgery [28]. Our study similarly showed no significant differences in PV and/or SMV occlusion at 6 months (primary repair 10.6%, end-to-end anastomosis 10.5%, interposition graft 20%, p = 0.390). However, their study did not consider mortality in patency calculation nor include any Kaplan-Meier estimates of venous patency.

Our institution also experienced excellent outcomes for patients with PTFE grafts with 100% patency (Table 4). The patency of PTFE graft appeared to be superior to vein allograft in our study unlike prior studies [15, 23], although this was not statistically significant. This is surprising as it is widely believed that synthetic grafts have increased risk of thrombosis due to the introduction of foreign graft material, resulting in an inflammatory reaction at the endothelial surface. Song et al. similarly demonstrated superior long-term patency of synthetic graft over autologous graft (synthetic graft OR: 2.14, 95% CI: 0.98–4.69, autologous graft OR: 3.20, 95% CI: 1.31–7.80) and postulated that autologous grafts may be associated with high fibrinogen and low protein C levels, predisposing to thrombosis [29]. The present study seems to support the hypothesis that synthetic grafts may have superior patency over autologous graft, but the apparent superior patency of PTFE graft could be due to the small sample size which may not reflect true differences. PTFE graft may also have an increased risk of infection due to direct seeding during implantation or haematogenous spread from a remote site [30]. Nonetheless, none of our patients experienced a graft infection with no abnormalities noted on routine CT surveillance scans. This is consistent with Liao et al. which reported no graft infections on 34 patients who underwent SMV-PV reconstruction [24].

Postoperative morbidity and mortality are also important considerations when comparing various types of venous reconstruction. Our study demonstrates no difference in incidence of morbidity which is consistent with existing literature [28]. Our findings were also concordant with a recent study by Terasaki et al. which demonstrated no significant differences in 30-day, 90-day, in-hospital mortality and Kaplan-Meier estimates between end-to-end anastomosis and interposition graft [28]. However, our median survival time for interposition graft was relatively shorter than the other venous reconstruction methods although not statistically significant (interposition graft 6.9 months, primary repair 13.8 months, end-to-end anastomosis 16.9 months). This is likely attributed to selection bias as patients who undergo interposition graft usually have more advanced disease and a larger tumour burden which warranted the need for an interposition graft. Additionally, the number of patients with interposition graft (n = 10/76(13.2%)) was relatively small.

Other perioperative outcomes were comparable between the various types of venous reconstruction, except for the need for blood transfusion and median operating time. Pairwise comparison with Bonferroni correction demonstrated increased blood transfusion for interposition graft over primary repair and end-to-end anastomosis (adjusted p = 0.037). Median operating time was significantly longer for interposition graft than primary repair (p = 0.001) and end-to-end anastomosis (p = 0.033). This is consistent with existing literature: Liao et al. demonstrated that interposition graft has longer operating duration and greater blood loss [24]. This was not surprising as interposition graft is usually used for more advanced tumors which require a more complicated and longer operation.

One of the limitations is the retrospective nature of the study with inherent selection bias. In addition, our total sample size of 76 patients was relatively small over a 13-year period. This is because it was only in the recent decade which demonstrated the safety of pancreaticoduodenectomy with venous reconstruction and its superiority over non-operative management [12]. We did not report the specific details of type of anti-platelets and/or anti-coagulants used in the study. As of current date, there is a lack of evidence on the type and duration of anti-coagulation used for patients with venous reconstruction and underlying malignancy and shows no significant differences in thrombosis rate between anti-coagulation and no anti-coagulation [15, 31]. Decision to start anti-coagulation was a shared decision between surgeon and patients.

## 5. Conclusions

Our study demonstrates superior 1-year patency of primary repair over end-to-end anastomosis and interposition graft with comparable morbidity and mortality. However, interposition graft should still be considered and used in situations where indicated, such as an extensive length of venous resection. Although this study adds to current literature on the patency of different venous reconstruction, large multi-centre trials are required to validate our findings.

## Supporting information

S1 File (SAV)
